# Effects of *Angelicae dahuricae Radix* on 2, 4-Dinitrochlorobenzene-Induced Atopic Dermatitis-Like Skin Lesions in mice model

**DOI:** 10.1186/s12906-017-1584-8

**Published:** 2017-02-07

**Authors:** Jin Mo Ku, Se Hyang Hong, Hyo In Kim, Hye Sook Seo, Yong Cheol Shin, Seong-Gyu Ko

**Affiliations:** 1 Department of Science in Korean Medicine, Graduate School, Kyung Hee University, Seoul, 02447 Korea; 2 Department of Preventive Medicine, College of Korean Medicine, Kyung Hee University, 1 Hoegi, Seoul, 130-701 Korea

**Keywords:** Atopic dermatitis, *Angelicae dahuricae Radix*, 2, 4-Dinitrocholrlbenzene, BALB/c mice, Cytokine, Inflammation

## Abstract

**Background:**

Atopic dermatitis (AD) is an inflammatory, chronically relapsing, and intensively pruritic skin disease that affect 10–30% of the global population. *Angelicae dahuricae Radix* (ADR) has been reported to be anti-inflammatory in Korean Medicine. In the present study, we investigated whether ADR suppresses the progression of AD in animal model.

**Methods:**

AD was induced by 2, 4-Dinitrochlorobenzene (DNCB). ADR was orally administered to mice to study the effect of ADR on AD. Histological Analysis, immunohistochemistry, blood analysis, RT-PCR, and ELISA assay were performed.

**Results:**

ADR significantly suppressed AD-like symptoms in BALB/c mice: ADR decreased skin thickness and spleen weight of mice. ADR reduced infiltration of mast cells, inflammatory cells and CD4+ cells into mouse skin. ADR lowered the number of WBCs in the blood of mice. ADR reduced the levels of IgE, IL-6, IL-10 and IL-12 in mice serum. ADR down-regulated mRNA expression of IL-4, IL-6 and TNF-α in mouse skin tissue.

**Conclusion:**

Our present study clearly indicates that ADR suppresses the progression of AD induced by DNCB in BALB/c mice. This suggests that ADR might be a useful drug for the treatment of AD.

## Background

10–30% of the global population in the world suffer by atopic dermatitis (AD), which is also known as atopic eczema, and is one of the most common allergic diseases [1, 2]. AD is characterized by chronic or relapsing skin disorder, skin barrier dysfunction, and pruritic skin inflammation [3–5]. 2–10% of adults and 15–30% of children suffer from AD experiencing a significant reduction in quality of life [6–8]. Over the past 10 years, the prevalence of AD has increased a two-fold in elementary school-aged children in South Korea [9]. Therefore, successful treatment of AD is a very important task to decline disease.

The pathogenesis of AD is not well known, but two main theories have been proposed. In one theory, it was explained that AD is associated with filaggrin gene mutations. Filaggrin is a filament-associated protein that binds to keratin fibers in epidermis. Defection in filaggrin seems to induce skin barrier dysfunction leading to water loss from the skin [10, 11]. The other theory is immunological hypothesis associated with Th1/Th2 imbalance. T-helper cells play an important role in disease onset and progression. There is a predominance of Th2 cells rather than Th1. This results in increased infiltration of inflammatory cells such as lymphocytes and macrophages into the skin lesions, and eosinophilia in peripheral blood. This also induces increased level of immunoglobulin E (IgE) [12, 13].

Surfaces of mast cells aggregate high-affinity IgE receptors (Fc*ε*I). IgE secretion is an important immediate hypersensitivity reaction in AD through mast cells. Mast cell activation releases not only inflammatory mediators but also Th2 cytokines (IL-4, IL-5 and IL-13) [14, 15]. Proinflammatory cytokines such as IL-6 and IL-10 play an important role in allergic inflammation [16, 17]. Moreover, proliferation of CD4+ T cells is observed in AD patient [18–20].

*Angelicae dahuricae Radix* (ADR) is a perennial plant that grows naturally. ADR are commonly known as Chinese Angelica, Wild Angelica, or Bai Zhi in Chinese [21]. ADR is known as Baig-Ji in Korean. ADR leaves are used to make strong scented incense. In addition, ADR are used in traditional medicine to counter harmful external influences on the skin, such as cold, headaches, rhinitis, heat, dampness and dryness [22]. In the experimental study, ADR alleviated the redness, swelling, and other symptoms of acute inflammation in mouse and rat [23]. ADR reduced the levels of the serum inflammatory mediators including Nitric oxide (NO), Tumor Necrosis Factor-alpha (TNF-α), and prostaglandid E2 (PGE2) [24]. ADR showed anti-inflammatory activity in RAW264.7 cell and cytotoxic effect in A549 cells and KB cells [25]. Main compound of ADR is known to be an aviprin [26]. Aviprin showed antioxidant activity and is cytotoxic on LNCaP and HeLa cell lines [27]. These results indicate that ADR may be good candidate for the control of AD and beneficial in the treatment of human allergic disorders.

In the present study, we investigated whether ADR oral administration has anti-inflammatory activity on 2,4-dinitrochlorobenzene-(DNCB-) induced AD-like skin lesions in mice model.

## Methods

### Preparation of *Angelicae dahuricae Radix* (ADR)

ADR was supplied by Han-poong Pharm Co., Ltd (Jeonjoo, Republic of Korea). ADR powder was dissolved in distilled water to give to mice a concentration of 200 mg/kg.

### Animals

Animal experiments were approved by Kyung Hee university institutional animal care of use committee (KHUASP(SE)) and performed according to ethical treatment. Six-week-old male BALB/c mice were purchased from Orient (Seoul, Republic of Korea). Mice were maintained for 1 week before the start of the experiment. Animals were randomized and all housed under controlled temperature (23 ± 3 °C) and humidity (55 ± 15%), with a 12 h light/12 h dark cycle. Animals were provided with a laboratory diet and water ad libitum. Body weight and Food intake of animals were measured once every 2 days.

### Induction of AD and treatment

**Fig. 1:**
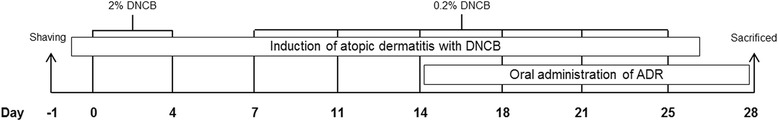
General schematic diagram of studies

Induction of AD procedure and treatment are described in Fig. 1. The mice were divided into three groups (*n* = 8): group 1, normal; group 2, DNCB; group 3, DNCB + ADR(200 mg/kg). For the experiment, mice back skin was painted dermally with 200 *μ* L of a 2% DNCB using 1 × 1 cm patches after shaving. Three weeks after sensitization, the back skin was challenged with 200 *μ*L of a 0.2% DNCB solution. Finally, mice were fed with ADR together with DNCB sensitiazation for 2 weeks. At the end of experiment, mice were sacrificed by CO_2_-inhalation, and samples were collected.

### Histological Analysis

Skin samples were embedded in Tissue-Tek optical cutting temperature (OCT) compound (Leica, USA). The section of the skin samples was 20 μm-thick. We used caliper to measure skin thickness. Each section was stained with hematoxylin and eosin (H & E) for inflammatory cells and with toluidine blue (T.B) for mast cells, and examined under light microscopy (Olympus). Mast cells and inflammatory cells were counted in 10 parts of high-power fields (HPF) (250 μm x 250 μm) at 40x, 400x and 1000x magnification.

### Immunohistochemistry

Expression of CD4+ lymphocytes was detected by immunohistochemical analysis using the anti-CD4+ antibody. The skin tissues were rehydrated. After a microwave treatment, the sections were treated with 3% hydrogen peroxide in PBS for 15 min to inhibit endogenous peroxidase activity of blood cells. The skin sections were blocked with 5% bovine serum albumin (BSA) in PBS for 1 h, at room temperature. Skin sections were incubated with mouse monoclonal CD4+ antibody overnight at 4 °C and subsequently incubated with secondary biotinylated anti-rabbit IgG for 1 h at room temperature. Sections were treated with avidin-biotin HRP complex (Vectastain ABC kit, Vector Labs, Burlingame, CA, USA) for 30 min at 4 °C and finally stained with diaminobenzidine tetrachloride (DAB) as a substrate. The slides were mounted with an aqueous mounting solution (DAKO, Glostrup, Denmark) and cover-slipped. All the sections were analyzed using an Olympus microscope and images were captured using a digital video camera.

### Analysis of mouse blood

Whole blood samples were collected by cardiac puncture. The blood was placed in Vacutainer TM tubes containing EDTA (BD science, Franklin Lakes, NJ, USA). Anti-coagulated blood was submitted to determination of hematological parameters (WBC, lymphocytes, monocytes, eosinophils, basophils and neutrophils) in a HEMAVET 950 hematology analyzer (Drew Scientific, Inc., Miami Lakes, FL, USA) in accordance to manufacturer’ recommendation.

### RT-PCR

RNA was isolated using easy-blue RNA extraction kit (iNtRON biotech, Seongnam, Republic of Korea) according to the manufacturer’s instructions. Isolated RNA content was measured using the NanoDrop ND-1000 spectrophotometer (NanoDrop Technologies Inc, Wilmington, North Carolina, USA). Total cellular RNA from each sample was reversely transcribed using cDNA synthesis kit (TaKaRa, Otsu, Shiga, Japan). PCR was performed using the specific primer. The primers used were as follows : mouse IL-4 (Forward : 5’ - TCG GCA TTT TGA ACG AGG TC - 3’, Reverse : 5’ - GAA AAG CCC GAA AGA GTC TC - 3’); mouse IL-6 (Forward : 5’ - GAT GCT ACC AAA CTG GAT ATA ATC - 3’, Reverse : 5’ - GGT CCT TAG CCA CTC CTT CTG TG - 3’); mouse TNF-α (Forward : 5’ - ATG AGC ACA GAA AGC ATG ATC - 3’, Reverse : 5’ - TAC AGG CTT GTC ACT GGA ATT - 3’); and mouse GAPDH (Forward : 5’ - GAG GGG CCA TCC ACA GTC TTC - 3’, Reverse : 5’ - CAT CAC CAT CTT CCA GGA GCG - 3’).

### Enzyme-Linked Immune Sorbent Assay (ELISA)

For measurement of total serum IgE, IL-6, IL-10 and IL-12, blood specimens were obtained from the heart on the final day. The blood was placed in Vacutainer tubes containing EDTA (BD science, Franklin Lakes, NJ, USA) and blood plasma was isolated. Total IgE, IL-6, IL-10 and IL-12 levels in plasma were determined by sandwich ELISA using the BD PharMingen ELISA set according to the manufacturer’s instruction. Optical densities were measured at 450 nm using a microplate reader (Versa Max, Molecular Devices, Sunnyvale, CA, USA).

### Statistical analysis

All quantitative data derived from this study were analyzed statistically. One-way ANOVA was used for analysis of our data. The results were expressed as the mean ± SEM. Statistical significance at *P* < 0.05 < 0.01 and < 0.001 has been given respective symbols in the figures.

## Results

### Effect of oral administration of ADR on body weight and food intake of mice

**Fig. 2:**
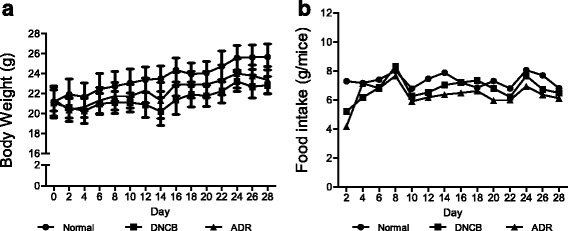
Changes in body weight (**a**) and Food intake (**b**) during treatment with ADR (200 mg/kg). After induction of AD by DNCB, mice were orally administered with ADR. Values are expressed as mean ± SEM (*n* = 8)

We monitored body weight and food intake of mice throughout the study. We observed no significant changes in this regard, indicating that ADR may not affect general conditions in mice (Fig. 2a and b).

### Effect of ADR on DNCB-induced AD in mice model

**Fig. 3:**
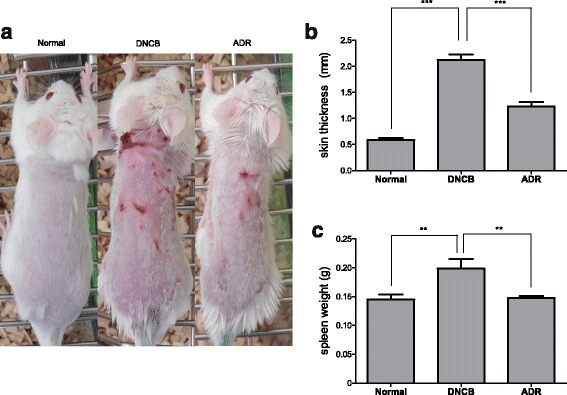
Observations of skin lesions in ADR (200 mg/kg) treated DNCB-induced AD mice. The photograph shows the back of mice on day 28 after sensitization (**a**). The measurement of skin thickness (**b**) spleen weight (**c**) in DNCB-induced AD mice model treated with ADR. Values are expressed as mean ± SEM (*n* = 8)

AD-like skin lesions were induced in Balb/c mice applying DNCB for 4 weeks. DNCB application was followed by ADR oral administration. ADR markedly inhibited AD as shown in Fig. 3a. A histogram of skin thickness estimated is shown in Fig. 3b. Normal skin was found to be 0.58 ± 0.12 mm (ranging from 0.46 to 0.84 mm), while DNCB-induced AD skin (negative control) was found to be 2.12 ± 0.27 mm (ranged from 1.92 to 2.66 mm). DNCB- induced AD skin orally treated with ADR was found to be 1.23 ± 0.21 mm (ranged from 0.92 to 1.48 mm). Data demonstrate that ADR decreased skin thickness of mice. Moreover, ADR decreased spleen weight of mice (Fig. 3b).

### Effect of ADR on mast cells, inflammatory cells and CD4+ cells

**Fig. 4:**
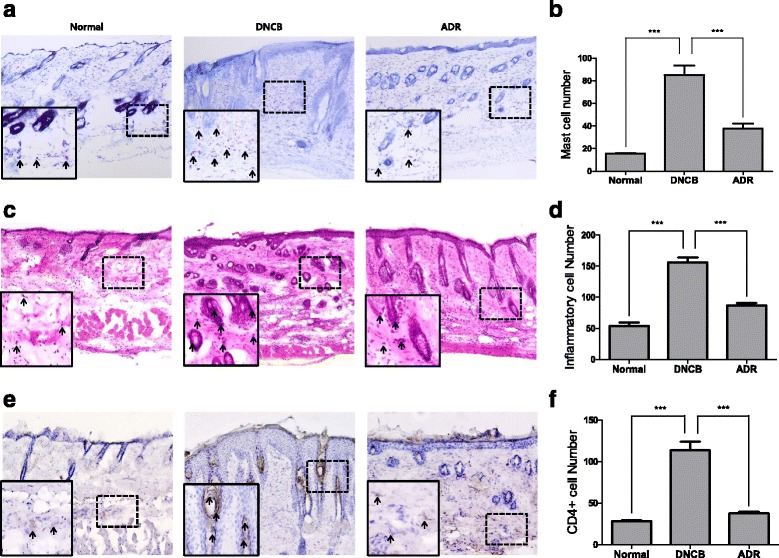
ADR (200 mg/kg) reduced infiltration of mast cells, inflammatory cells and CD4+ cells into skin. The skin sections were stained with toluidine blue (**a**). Sections were evaluated using microscope at an original magnification of 200x. The skin sections were stained with hematoxylin and eosin (**c**). Sections were evaluated using microscope at an original magnification of 200x. The skin sections were immunostained with CD4+ antibody. CD4+ cells were shown as brown color (**e**). **b**, **d**, **f** Sections were evaluated and graphed from the results of (**a**), (**c**), (**e**). Sections were evaluated using microscope at an original magnifiation of 1000x. Data are presented as mean ± SEM. ∗*P*< 0.05, ∗∗*P*< 0.01, and ∗∗∗ < 0.001

To determine whether ADR reduces infiltration of mast cells and inflammatory cells into skin, we performed T & B staining and H & E staining on the skin samples. Numbers of mast cells and inflammatory cells in AD mice were shown to be higher than those in normal mice. ADR decreased such infiltration of mast cells and inflammatory cells into skin (Fig. 4a, c). Mast cells and inflammatory cells numbers under each condition were shown in Fig. 4b, d. We performed immunocytochemistry to examine whether ADR reduces level of CD4+ (total T cells) within skin. The level of CD4+ in DNCB-induced AD lesions in mice is higher than that in normal mice. ADR decreased the level of CD4+ cells within the skin (Fig. 4e, f).

### Effect of ADR on WBCs in the blood of mice

**Fig. 5:**
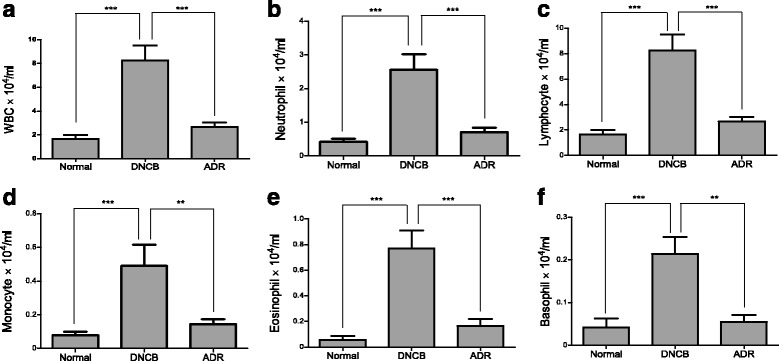
ADR (200 mg/kg) reduced the levels of leukocytes in the blood. WBC (**a**), Neutrophil (**b**), Lymphocyte (**c**), Monocyte (**d**), Eosinophil (**e**), Basophil (**f**) were analyzed using HEMAVET 950 hematology analyzer. Data are presented as mean ± SEM. ∗*P*< 0.05, ∗∗*P*< 0.01, and ∗∗∗*P*< 0.001

To investigate whether ADR suppresses inflammatory phenomenon, we measured leukocytes levels in cardiovascular blood samples using HEMAVET 950 hematology analyzer. We observed that DNCB application increased total number of white blood cells (WBCs) and each subtypes of WBCs including neutrophils, basophils, eosinophils, monocytes, and lymphocytes in serum of mice. Importantly, a subsequent oral administration of ADR lowered the increased number of WBCs, implicating ADR suppresses inflammatory responses by decreasing the number of WBCs in the blood (Fig. 5a, b, c, d, e and f).

### Effect of ADR on the levels of IgE, IL-6, IL-10 and IL-12 in mice serum

We measured the levels of inflammatory cytokines in the blood samples by ELISA assay.

**Fig. 6:**
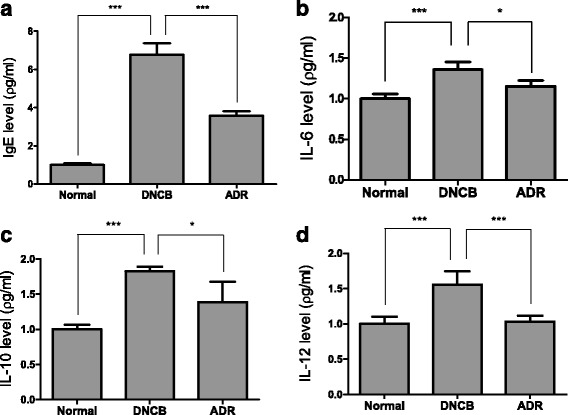
ADR (200 mg/kg) reduced the levels of cytokines in the serum. The release of IgE (**a**), IL-6 (**b**), IL-10 (**c**) and IL-12 (**d**) was measured by sandwich ELISA assay. Data are presented as mean ± SEM. ∗*P*< 0.05, ∗∗*P*< 0.01, and ∗∗∗*P*< 0.001

We found that DNCB increased the levels of IgE, IL-6, IL-10 and IL-12 while ADR inhibited such increases (Fig. 6a, b, c and d).

### Effect of ADR on mRNA expression of IL-4, IL-6 and TNF-α in mouse skin tissue

**Fig. 7:**
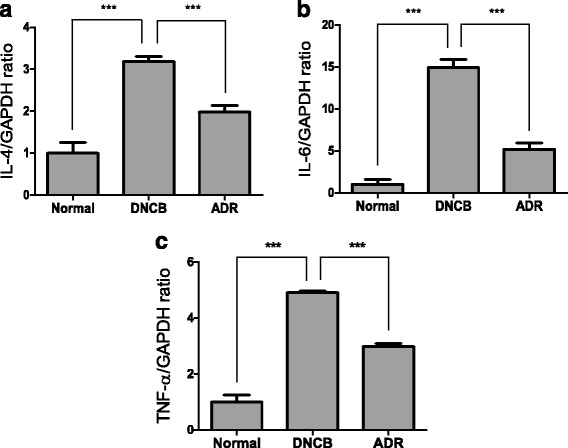
Effect of ADR (200 mg/kg) on the cytokine mRNA expression in mouse skin tissue. The IL-4, IL-6 and TNF-α mRNA expression were measured by RT-PCR (**a**), (**b**), (**c**) in mouse skin tissue. The columns and the error bars represent mean ± SD (*n* = 8 mice/group). ∗*P*< 0.05, ∗∗*P*< 0.01, and ∗∗∗ < 0.001

To determine whether ADR decreases AD-relevant cytokines expression, we performed RT-PCR to measure levels of IL-4, IL-6 and TNF-α. We found that DNCB increased the levels of IL-4, IL-6 and TNF-α while ADR decreased such increases (Fig. 7a, b, c and d).

## Discussion

AD is a chronic inflammatory skin disease, which increases serum immunoglobulin E (IgE) levels and infiltration of inflammatory cellincluding mast cells and eosinophils [28, 29]. The pathogenesis of AD is primarily driven by Th2 immune responses [14]. This causes epidermal thickness of mice with cutaneous hypersensitivity.

CD4+ T cells and mast cells are known as key factors in allergic inflammatory diseases. AD mouse model contains increased CD4+ T cell [30, 31]. DNCB-induced AD mouse model showed severe AD symptoms, increase in mast cells, epidermal hyperplasia and elevation of serum IgE levels. DNCB-induced BALB/c mice model represented increase of IL-4 mRNA level. BALB/c mouse is advantageous AD model as compared to other animal model since it develops Th2-skewed immune response [32, 33].

In this study, we investigated the anti-AD effects of ADR using DNCB-applied BALB/c mice. We found that oral administration of ADR strongly suppressed DNCB-induced AD-like symptoms such as skin thickness. Normal skin was found to be 0.58 ± 0.12 mm (ranging from 0.46 to 0.84 mm), while DNCB-induced AD skin (negative control) was found to be 2.12 ± 0.27 mm (ranged from 1.92 to 2.66 mm). DNCB- induced AD skin orally treated with ADR was found to be 1.23 ± 0.21 mm (ranged from 0.92 to 1.48 mm). ADR also reduced infiltration of mast cells, inflammatory cells and CD4+ cells into the sensitized skin. Numbers of mast cells and inflammatory cells in AD mice were shown to be higher than those in normal mice. ADR decreased such infiltration of mast cells and inflammatory cells into skin. The level of CD4+ in DNCB-induced AD lesions in mice is higher than that in normal mice. ADR decreased the level of CD4+ cells within the skin

In AD skin, activated Th2 cells would produce IgE by releasing cytokines such as IL-4 [34, 35]. Associated Th2 inflammatory cytokine, such as IL-4 and IL-6 could promote the occurrence and development of inflammatory reactions [19, 36, 37]. In our study, ADR application decreased the serum levels of IgE, IL-6, IL-10 and IL-12 that are induced by DNCB treatment. DNCB increased the levels of IgE, IL-6, IL-10 and IL-12 while ADR inhibited such increases. ADR application reduced the DNCB-stimulated increases of the number of eosinophils, neutrophils, monocytes, basophils, lymphocytes and WBC. A subsequent oral administration of ADR lowered the increased number of WBCs, implicating ADR suppresses inflammatory responses by decreasing the number of WBCs in the blood Moreover, ADR reduced the levels of IL-4, IL-6 and TNF-α mRNA expression. ADR exhibited decrease in mast cell recruitment and serum IgE levels. ADR reduced infiltration of CD4+ cells into mouse skin. ADR suppressed the expression of inflammatory cytokine including IL-4, IL-6, IL-10 and TNF-α. DNCB increased the levels of IL-4, IL-6 and TNF-α while ADR decreased such increases. These results suggest that ADR suppresses skin inflammation by inhibiting the DNCB-stimulated numerous inflammatory responses. Moreover, free radicals are unstable and independent but can be cause of many diseases including cancer and atopic dermatitis. ADR seems to act against free radicals by removing them [38, 39] Limitations of AD model is that animal study generates many differences from human study in terms of the anatomical, physiological, and immunological contributors. Moreover, it is difficult to establish chronic disease model in mice. Nevertheless, AD mice model is still important tool to investigate chronic disease for human being.

## Conclusions

Our present study clearly demonstrates that ADR suppresses the progression of AD induced by DNCB. ADR significantly suppressed AD-like symptoms in BALB/c mice: ADR decreased skin thickness and spleen weight of mice. ADR reduced infiltration of mast cells, inflammatory cells and CD4+ cells into mouse skin. ADR lowered the number of WBCs in the blood of mice. ADR reduced the levels of IgE, IL-6, IL-10 and IL-12 in mice serum. ADR down-regulated mRNA expression of IL-4, IL-6 and TNF-α in mouse skin tissue. Therefore, ADR might be a useful drug for the treatment of AD. Our data indicates that ADR have a potential as a new drug for the suppression of AD.
